# Beyond the Bench: Fish Tales to Ensure Health

**DOI:** 10.1289/ehp.112-a738

**Published:** 2004-09

**Authors:** Kimberly G. Thigpen, David Petering

Fishermen are known for telling tales of their catches that tend toward exaggeration. A new kind of fish tale, however, doesn’t stretch the truth when making a point to the Hmong community in Milwaukee, Wisconsin, about the hazards of eating fish contaminated with methylmercury and polychlorinated biphenyls. A video produced by the Community Outreach and Education Program at the NIEHS Marine and Freshwater Biomedical Sciences Center at the University of Wisconsin–Milwaukee communicates in a simple, understandable, and culturally sensitive way the risks of eating contaminated fish and teaches methods of catching and preparing fish that can reduce these risks.

The Hmong, refugees from Southeast Asia, are avid anglers and traditionally fish to support large families with an average of 7–8 children. But these fishers often have little understanding of the pollution and contamination of the waterways of Wisconsin and the fish that populate them. The goal of the center program, developed in partnership with the Hmong/American Friendship Association and the Sixteenth Street Community Health Center, is to communicate to the inner-city Hmong population the hazards associated with eating contaminated fish in a way that results in active consideration of the issues within the context of the group’s fishing practices.

The centerpiece of the outreach program is a bilingual Hmong–English video titled *Nyob Paug Hauv Qab Thu* (*Below the Surface*). The video presents scientifically sound information on safe fish consumption. It also acknowledges the Hmong cultural tradition of fishing while showing which fish are safest to catch and ways to make fish safer to eat prior to cooking, including how to remove fins, fat, and other parts of the fish where toxicants accumulate. The video is packaged with a laminated card that provides shorthand tips on safer catching and preparation, and a kitchen magnet with similar information.

To date, approximately 750 video/card/magnet packets have been distributed by community workers through local stores, doctors’ offices, and Hmong festivals where the video has been showcased. Follow-up to assess the impact of the videos is currently under way.

John Dellinger, a center researcher who studies the effects of fish consumption in Native American populations and who is featured in the film, has also shown the video or supplemental materials to audiences of Intertribal Council and InterTribal Fisheries Assessment Program officials in the Upper Peninsula of Michigan, as well as to Tahitian Ministry of Health officials. Officials of the Michigan Ojibwa and the government of Tahiti have asked that the film be adapted for their communities. Dellinger plans to work on productions for both of these groups in 2005.

In an extension of the community outreach program, the center has developed a life sciences classroom module for middle-school students that explores the behavioral effects of mercury and lead contamination, both of which affect inner-city Hmong populations. The module provides a hands-on, inquiry-based experiment about a complex organism’s behavioral integration with its environment, and what happens when that environment becomes contaminated.

In the module, students observe fathead minnows in the classroom to learn and characterize their normal reproductive behavior. Students then watch a video produced by the center that shows the behavior of mercury- and lead-exposed fish. Based on their understanding of normal behavior, students analyze the differences that exposure to the toxic metals makes in the fish. The differences are dramatic because although the behavior is affected, the fish show no outward physiological signs of toxicity. Teachers can then draw an analogy to human exposure to mercury through fish consumption, and to lead through paint chip ingestion, and the potential resulting effects on human behavior.

Teachers were trained during a summer workshop to teach and evaluate the experiment. In the next two years, 11 teachers from Milwaukee public schools and from area suburban schools with the largest Hmong student populations will introduce this module, using the video as a cross-cultural tool to support it.

## Figures and Tables

**Figure:**
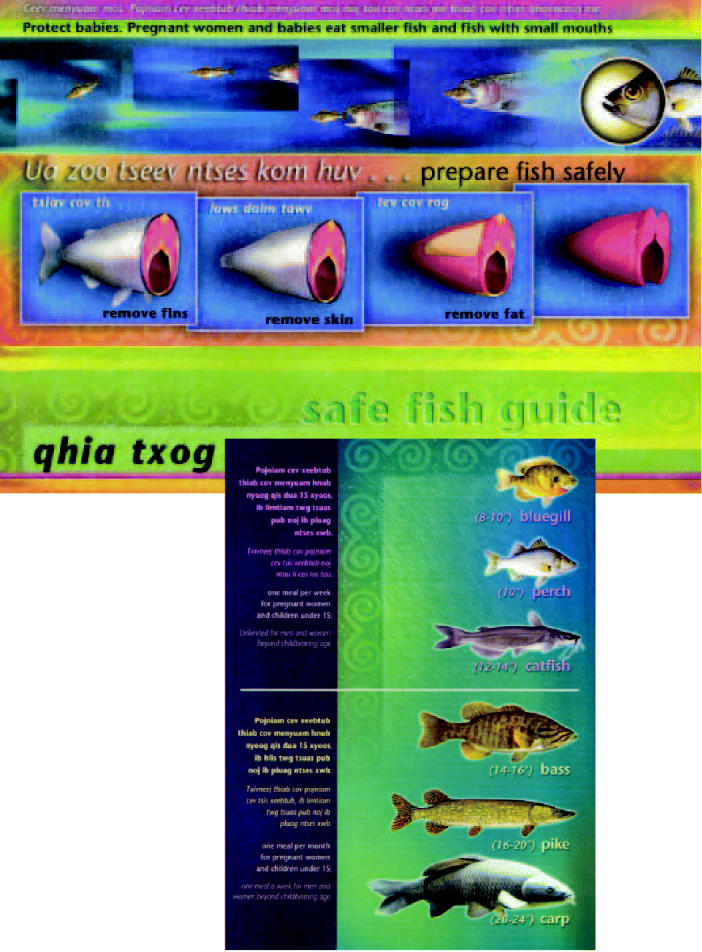
**New tool for the tacklebox.** Laminated cards with fish safety information are distributed to Hmong anglers.

**Figure:**
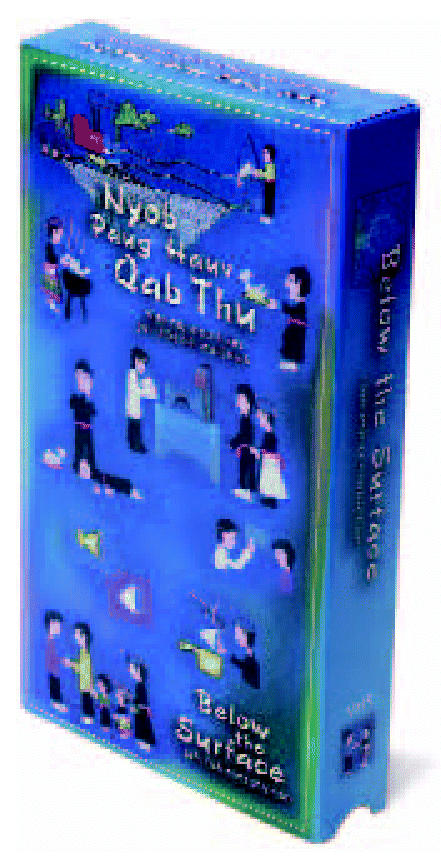
**Translating into health.** A bilingual video educates Hmong fishers on health risks and safety measures.

